# Computational Evaluation of Abrogation of HBx-Bcl-xL Complex with High-Affinity Carbon Nanotubes (Fullerene) to Halt the Hepatitis B Virus Replication

**DOI:** 10.3390/molecules26216433

**Published:** 2021-10-25

**Authors:** Abbas Khan, Omar Ahsan, Dong-Qing Wei, Jawad Khaliq Ansari, Muzammil Hasan Najmi, Khalid Muhammad, Yasir Waheed

**Affiliations:** 1 Foundation University Medical College, Foundation University Islamabad, DHA-I, Islamabad 44000, Pakistan; biotechuos4@gmail.com (A.K.); omarahsan71@gmail.com (O.A.); dr.jkansari@gmail.com (J.K.A.); najmimh@hotmail.com (M.H.N.); 2 Department of Bioinformatics and Biological Statistics, School of Life Sciences and Biotechnology, Shanghai Jiao Tong University, Shanghai 200240, China; dqwei@sjtu.edu.cn; 3 State Key Laboratory of Microbial Metabolism, Shanghai-Islamabad-Belgrade Joint Innovation Centre on Antibacterial Resistances, Joint Laboratory of International Laboratory of Metabolic and Developmental Sciences, Ministry of Education and School of Life Sciences and Biotechnology, Shanghai Jiao Tong University, Shanghai 200030, China; 4 Peng Cheng Laboratory, Vanke Cloud City Phase I Building 8, Xili Street, Nashan District, Shenzhen 518055, China; 5 Department of Biology, College of Sciences, United Arab Emirates University, Al Ain 15551, United Arab Emirates

**Keywords:** HBV, carbon nanotubes, docking, IFD, simulation, free energy calculation

## Abstract

Hepatitis B virus (HBV) is the world’s most prevalent chronic viral infection. More than 350 million individuals are chronic carriers of the virus, with an estimated 2 billion infected persons. For instance, the role of HBx protein in attachment and infection is very obvious and consequently deemed as an important druggable target. Targeting the interface and discovering novel drugs greatly advanced the field of therapeutics development. Therefore, in the current study, HBx to Bcl-xL is abrogated on high-affinity carbon nanotubes using computational structural biology tools. Our analysis revealed that among the total 62 carbon fullerenes, only 13 compounds exhibited inhibitory activity against HBx, which was further confirmed through IFD-based rescoring. Structural dynamics investigation revealed stable binding, compactness, and hydrogen bonds reprogramming. Moreover, the binding free energy calculation results revealed that the top hits1-4 possess the total binding energy of −54.36 kcal/mol (hit1), −50.81 kcal/mol (hit2), −47.09 kcal/mol (hit3), and −45.59 kcal/mol for hit4. In addition, the predicted K_D_ values and bioactivity scores further validated the inhibitory potential of these top hits. The identified compounds need further in vitro and in vivo validation to aid the treatment process of HBV.

## 1. Introduction

Hepatitis B virus (HBV) is the world’s most prevalent chronic viral infection. More than 350 million individuals are chronic carriers of the virus, with an estimated 2 billion infected persons. One HBV infection was recognized as the 10th largest cause of mortality in the 2010 Global Burden of Disease study and was the 10th leading cause of death (786,000 deaths per year) [1]. As a result of these findings, WHO has made viral hepatitis one of its top public health concerns. 

HBV is a small DNA virus with a genomic size of about 3.2 kb [2]. Hepatitis and hepatocellular carcinoma (HCC), a significant cause of liver cancer globally, are triggered by HBV infection. The HBV genome generates four viral gene transcripts, one of which, the HBV X protein (HBx), comes into contact with a number of host proteins and is required for HBV survival [3,4]. For example, HBx has been found to interact with DDB1, which might induce the DDB1-containing E3 ubiquitin ligase to attack the structural integrity of the chromosome 5/6 complex (Smc5/6) for degradation, liberating Smc5/6’s transcriptional control and augmenting HBV viral transcriptional activity [5,6]. HBV DNA fragments are incorporated into several locations of the host genome, and the HBx gene is often overexpressed in the livers and tumors of HBV chronic carriers. As a result, HBx plays a role in the development of chronic liver disease and HCC [7]. Recently, it was discovered that HBx uses a Bcl-2 homology region 3 (BH3)-like motif to directly target antiapoptotic proteins Bcl-2 and Bcl-xL, causing an increase in cytosolic calcium, which is required for HBV viral replication, as well as cytotoxic effects through apoptosis and necrosis, leading to HBV pathogenesis [8]. Despite the recent publication of the structure of the HBx-BH3-like peptide/Bcl-2 complex, the structural basis of the interaction between the HBx-BH3-like motif and the Bcl-xL protein, as well as the impact of this contact on the HBV life cycle have yet to be identified [9].

The HBx-BH3-like motif, which is distantly related to the canonical BH3-only motif, produces an unusual short amphipathic helix that fits into a unique hydrophobic pocket in Bcl-xL, according to research [10]. The HBx-BH3-like motif connects to a hydrophobic pocket 2 away from the conventional pocket in Bcl-xL that mediates binding to other BH3-only domains, unlike previous reported structures of Bcl-xL in combination with BH3-only-containing proteins [11]. Trp120 and Leu123, two residues in the HBx-BH3-like motif, engage hydrophobically with residues in the Bcl-xL binding pocket. The ability of a peptide containing the HBx-BH3-like motif (HBx-BH3-aa113–135) to pull down the native Bcl-xL protein in HepG2 cells, but not an analogous peptide with the W120A/L123A double mutations, supports this structural observation [12]. More significantly, this HBx-BH3-like peptide can restore HBV reproduction and transcription in HepG2 cells transfected with a replication-defective HBx-null HBV replicon but not an analogous peptide with the W120A/L123A double mutations [8,12]. The role of HBx protein in attachment and infection is very obvious and consequently deemed as an important druggable target. Targeting the interface and discovering novel drugs greatly advanced the field of therapeutics development. Therefore, in the current study, HBx to Bcl-xL is abrogated on high-affinity carbon nanotubes using computational structural biology tools. Using molecular docking and molecular dynamics simulation, we target the interface of HBX-BLC-XL to curtail the role of this complex in infection.

## 2. Computational Experiments

### 2.1. Protein Modelling, Carbon Nanotubes Retrieval, and Preparation

The structure of HBx is not yet available, hence a computational modelling approach was employed to model the 3-D structure of HBx. For this purpose, Robetta webserver was used using the ab initio modelling approach because of the nonavailability of templates [13]. The modelled structure was then subjected to energy minimization and refinement using GalaxyRefine webserver [14]. For structural quality evaluation, ProSA-web and Rampage servers were used [15].

### 2.2. Screening of Carbon Nanotubes against the HBx-Bcl-xL Interface

To identify potential inhibitors of the HBx-Bcl-xL complex, a literature search was carried out and 62 different carbon nanotubes collected [16]. These nanotubes were prepared and minimized using PyRx tool [17]. All these nanotubes were then screened against the binding interface of HBx-Bcl-xL. The residues 113–135 of HBx are reported to be responsible for communication with Bcl-xL. So, these residues were selected as the binding site residues. Based on the scoring, the best hits were selected for redocking and rescoring.

### 2.3. Induced-Fit Docking (IFD) of the Top Hits

In the next round, screening of the best scoring compounds, IFD (induced-fit docking) was carried out using 64 exhaustiveness to confirm the final hits. For IFD, AutoDockFR–AutoDock for Flexible Receptors (ADFR) [18], which uses the AutoDock4 scoring function to down-weight the receptor internal energy and handles the receptor sidechain conformational optimization of up to 14 different sidechains to enhance the success rate of docking, was used. AutoDockFR achieved higher accuracy than AutoDock Vina in cross-validation docking and also the speed of docking was much higher.

### 2.4. Molecular Dynamics Simulation of the Top Hits

All-atoms MD simulation of the top hits from the screening was performed using Amber18 package [19]. For drug topologies, the antechamber module was used, while The Amber general force field (GAFF) and ff14SB force fields were employed for the complex simulations. A TIP3P box of water and Na+ counter ions were used to solvate and neutralize each system subsequently. The energy minimization of the systems was carried out in two stages, followed by heating and equilibration. The Particle Mesh Ewald (PME) algorithm was used to quantify long-range electrostatic interactions [20]. A 1.4 nm cut-off value was set for Van der Waals interactions, as well as for Columbic interactions of short range. The Langevin thermostat was employed at a temperature constant at 300 K whereas for pressure control, the Berendsen barostat was considered. A time step of 2 fs and total simulation time of 50 ns for each complex was performed. The dynamics, stability, and other features of the ligand-protein complexes were evaluated by using CPPTRAJ and PTRAJ [21].

### 2.5. The Binding Free Energy Calculation

For all protein-ligand complexes, the free binding energy was calculated using the script MMPBSA.PY by considering 2500 snapshots using the following equation [22,23,24,25]. Different studies widely use this free energy calculation method to estimate TBE of different ligands [26,27]:

ΔG_bind_ = ΔG_complex_ − [ΔG_receptor_ + ΔG_ligand_]

where ΔG_bind_ denotes the total free binding energy, while others denote the free energy of the protein, ligand, and complex. The following equation was used to calculate the specific energy term contribution to the total free energy:

G = G_bond_ + G_ele_ + G_vdW_ + G_pol_ + G_npol_

Bonded, electrostatic, polar, non-polar, and van der Waal energy terms are represented by the above equation.

### 2.6. Dissociation Contant (K_D_) and Bioactivity Prediction

Prediction of protein-ligand binding affinity is largely calculated using dissociation constant and has been widely practiced by various studies [28,29,30]. Herein, we employed the same approach using PRODIGY webserver to predict the binding strength [31]. Moreover, we also predicted the bioactivity using Molinspiration to further validate our findings.

## 3. Results and Discussion

### 3.1. Structural Modelling and Evaluation

**Figure 1:**
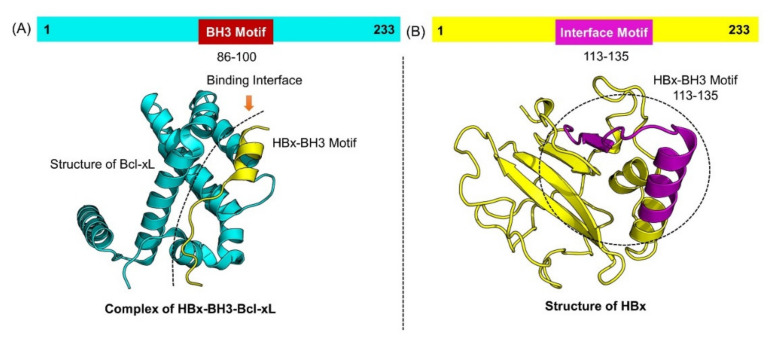
(**A**) Structure of Bcl-xL in complex with the interacting motif from HBx shown in cyan and magenta color. (**B**) Represents the modeled structure of HBx while the interface motif is shown as magenta.

HBx is a small 154 amino acid viral protein, which is involved in many cellular processes during hepatitis infection. This protein has been reported to interact with many host proteins and performs various processes, such as cell cycle progression, signaling, protein degradation, and chromosomal instability. Recently, a study reported that the interaction of HBx with the host Bcl-xL may assist the viral replication and survival inside the host cell. They reported that the BH3 motif of Bcl-xL interacts with aa113–135 to alter the cellular processes (Figure 1A,B). Therefore, due to its crucial role in various cellular processes and interaction with different host proteins, HBx is deemed as an important drug target. Consequently, in the current study, a structural modelling approach was employed to design novel potent nanomedicine-based inhibitors for the treatment of HBV. Since the complete structure of HBx is not yet available, the structure of HBx was modelled using Robetta server using an initio modelling approach. The structure was prepared and minimized prior to screening of carbon nanotubes. ProSA-Web server revealed a Z-score of −6.5 and Ramachandran plot revealed that 98% of the total residues lie in the favorable region, 1.1% in the allowed while only 0.9% residues were reported in the disallowed region. This shows that the structure is modeled well and could be used for further processes.

### 3.2. Screening of Carbon Nanotubes against the Interface of HBx-Bcl-xL

For screening, a total of 62 carbon nanotubes (fullerenes) were collected from different literature and screened against the interface of the HBx-BH3-Bcl-xL using the PyRx virtual screening tool. Ligands were prepared prior to docking and the binding residues aa113–135 were defined. A two-step scoring approach was employed. In the first step, all 62 carbon fullerenes were screened, for which the score range was −6.85 to −4.32 kcal/mol. In the next round, screening of the best 13 scoring compounds using IFD (induced-fit docking) was carried out using 64 exhaustiveness to confirm the final hits. For IFD, AutoDockFR–AutoDock for Flexible Receptors (ADFR) [18], which uses the AutoDock4 scoring function to down-weight the receptor internal energy and handles the receptor sidechain conformational optimization up to 14 different sidechains to enhance the success rate of docking, was used. 

**Figure 2:**
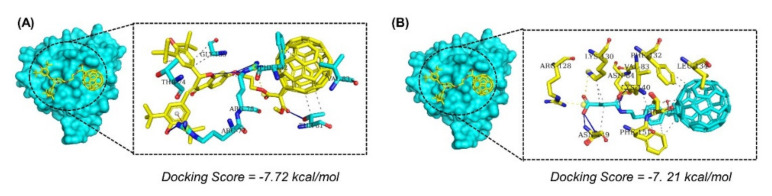
(**A**) Binding mode of the best hit 1. The best hit is shown as yellow while the interacting amino acids are shown in cyan color. (**B**) Binding mode of the best hit 2. The best hit is shown as yellow while the interacting amino acids are shown in cyan color.

AutoDockFR achieved higher accuracy than AutoDock Vina in cross-validation docking, and the speed of docking was much higher. A manual threshold of >−7.0 kcal/mol was set to select the best hits for further analysis. The IFD docking with the above criteria yielded only 4 compounds with scores > −7.0 kcal/mol. The docking score for the first two best compounds was reported to be −7.72 and −7.21 kcal/mol, respectively. The compound 1 (Figure 2A) established seven hydrogen bonds and nine hydrophobic interactions while two salt bridges were also observed. Arg77, Arg78, and Thr81 were involved in hydrogen bonding and Phe132 and Gly135 were involved in salt bridges. The hydrophobic interactions were formed between the ligand and multiple amino acids of the binding interface. On the other hand, compound 2 (Figure 2B) established six hydrogen bonds, three salt bridges, and multiple hydrophobic interactions. The binding patterns of compounds 1 and 2 are shown in Figure 2.

**Figure 3:**
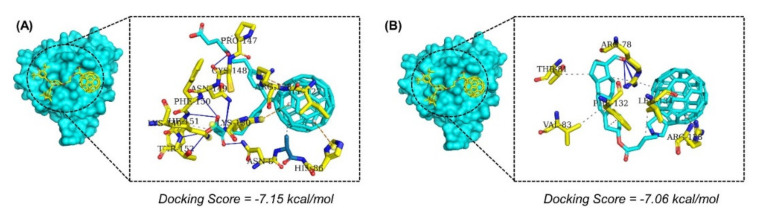
(**A**) Binding mode of the best hit 3. The best hit is shown as yellow while the interacting amino acids are shown in cyan color. (**B**) Binding mode of the best hit 4. The best hit is shown as yellow while the interacting amino acids are shown in cyan color.

In addition, with the docking score of −7.15 kcal/mol, he best hit 3 revealed a similar pattern of interaction with the interface amino acids. With six hydrogen bonds and various salt bridges, the compound was ranked among the best hits identified through molecular search. Moreover, the best hit 4 with three hydrogen bonds only and various hydrophobic interactions occupied the binding interface. The docking score for the best hit 3 was reported to be −7.06 kcal/mol. The interaction patterns of the best hits 3 and 4 are shown in Figure 3A,B.

### 3.3. Structural Stability Evaluation

**Figure 4:**
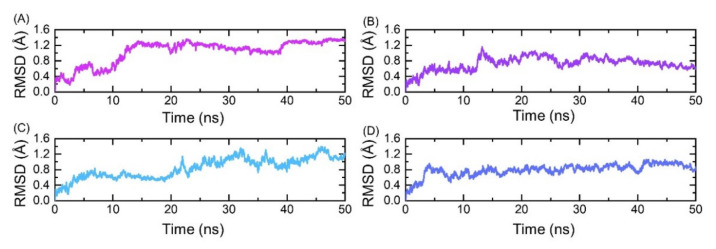
(**A**) Root mean square deviation of the best hit 1. (**B**) Root mean square deviation of the best hit 2. (**C**) Root mean square deviation of the best hit 3. (**D**) Root mean square deviation of the best hit 4.

Evaluation of the structural stability in a dynamic environment is an important parameter to estimate the binding stability inside the pocket. The dynamic stability of the top four hits was calculated and is shown in Figure 4A–D. The results revealed that the structures exhibit stable dynamic behavior during the 50 ns simulation time period except the best hit 1, which showed a little instable behavior during the first 15 ns. Initially, the RMSD gradually increased until 15 ns with many fluctuations at different time intervals; however, the system reached equilibrium and the graph became flatter until 50 ns, except a minor deviation at 38 ns was observed. On contrary, the dynamic behavior of the top hit 2 was more stable than hit 1. The RMSD soon reached the equilibrium point at 3 ns and then became flatter. A minor deviation between 12 and 15 ns was observed, where the RMSD increased up to 0.8 Å. The stability graph of the best hit 3 reflects a similar behavior to hit 1. The RMSD gradually increased with time; however, the RMSD value remained lower than required. The RMSD pattern of the best hit 4 was more comparable to the best hit 2. The mean RMSDs for all complexes were reported to be 0.6, 0.4, 0.6, and 0.4 Å, respectively. Conclusively, this shows that the top hits 1–4 possessed stable dynamics and could robustly interact with the interface residue to curtail the binding of Bcl-xL to the HBx.

### 3.4. Structural Compactness Evaluation

**Figure 5:**
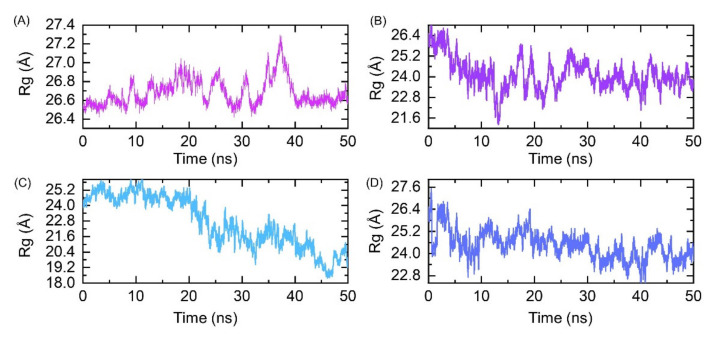
(**A**) Rg of the best hit 1. (**B**) Rg of the best hit 2. (**C**) Rg of the best hit 3. (**D**) Rg of the best hit 4.

The radius of gyration (*Rg*) was calculated to evaluate the compactness during the simulation, as given in Figure 5A–D. It is considered as an important parameter to estimate the binding stability inside the cavity. From Figure 5A, it can be seen that until 30 ns, the structure of the best hit 1 remained compact with few minor fluctuations, while during the last 20 ns, the compactness was lost and particularly between 35 and 40 ns the Rg value was 27.2 Å. During the last 10 ns, the Rg value significantly decreased, thus showing the stable binding of the best hit 1 to the interface residues. On the other hand, the structure of hit 2 remained more compact than hit 1. The Rg value for the first 15 ns remained 25.0 Å, and then decreased to 23.0 Å until 50 ns. Similarly, the structural compactness of the best hits 3–4 was also observed to be comparable to hit 2. The Rg value decreased during the simulation and remained lower until the end of the simulation. This shows that the increase or decrease in the Rg is due to the binding and unbinding of the bound ligands to the receptor. Decisively, this shows that the top hits 1–4 stably bind to the receptor and thus possess strong pharmacological activity against HBx.

### 3.5. Evaluation of Residual Flexibility

**Figure 6:**
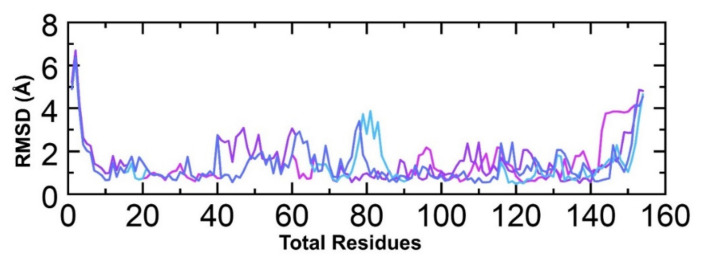
Root mean square fluctuation of all hits. The regions between 40 and 80 and then 105 and 140 particularly exhibit variations in the fluctuations. The region of 105–140 is the ligand binding site, thus implying that the binding of the ligands produces different conformational dynamics.

Understanding the residue level flexibility of the system is key to highlighting residues that are vital in holding the interacting ligand and overall stabilization of the complex. As can be seen in Figure 6, the majority of residues of the systems are in a significant equilibrium state with a mean RMSF of around 2.5 Å. A more similar pattern of RMSF for each complex was observed. The regions between 40 and 80 and then 105 and 140 particularly exhibited variations in the fluctuations. The region of 105–140 is the ligand binding site, thus implying that the binding of the ligands produces different conformational dynamics. Conclusively, the difference in the dynamic flexibility results in variable conformational optimization and binding to HBx.

### 3.6. Estimation of Binding Free Energy

The strength of a biomolecular association can be determined by estimating the binding affinity of the two interacting macromolecules. Computations of binding free energy using MM/GBSA methods are the most commonly used approach to rerank docking conformations via calculations of the structural-dynamic stability, the strength of interacting key hotspots, and total binding energies. The aforesaid method is computationally inexpensive compared to any other method, i.e., alchemical free energy calculation method. The MM/GBSA technique is considered as more accurate and comprehensive than the conventional scoring functions. Thus, to reevaluate the binding scores of the best complexes, we employed the MM/GBSA approach using 2500 structural frames (Table 1). The Van Der Waal energy for each complex (hit 1–4) was reported to be −162.51, −156.32, −141.11, and −158.42 kcal/mol, respectively. On the other hand, the electrostatic energy for each complex was reported to be −193.89, −189.47, −202.12, and −196.80 kcal/mol. The total binding energy for the top hit 1 was −54.36 kcal/mol, −50.81 kcal/mol for hit 2, −47.09 kcal/mol for hit 3, and −45.59 kcal/mol for hit 4, respectively. In conclusion, hit 1 exhibited stronger binding energy followed by hit 2, while hit 3 and hit 4 were ranked after hit 1 and hit 2. The results show that all compounds obtained from the molecular search exhibit stronger binding affinity, and thus strong inhibitory and pharmacological activity against HBx.

### 3.7. Dissociation Constant and Bioactivity Prediction

The KD results revealed that compound 1 has a KD value of −9.4 kcal/mol while the bioactivity score was 0.36. The KD value for compounds **1**–**3** was reported to be −8.63, −8.26, and −7.93 kcal/mol, respectively, which shows stronger binding of these compounds to the interface and stronger inhibitory properties. Molinspiration predicted the bioactivity scores for compounds **1**–**3** were 0.25, −0.12, and 0.19, respectively. The scores between −0.5 and 0.5 are regarded as the best bioactivity scores against different classes of drug targets.

## 4. Conclusions

In conclusion, the current study used molecular modeling approaches, which identified the four best hits from the in house-built database of carbon fullerenes. The shortlisted compounds blocked the interface of HBx-BcL and consequently assured the unavailability of the interfaces for binding, thus curtailing the interaction required for infection.

## Figures and Tables

**Table 1:** Binding free energy calculated as MM/GBSA. All energies are given in kcal/mol.

Complex Name	VDW	ELE	GB	SA	Total
Complex 1	−162.51	−193.89	320.95	−18.91	−54.36
Complex 2	−156.32	−189.47	312.21	−17.23	−50.81
Complex 3	−141.11	−202.12	315.25	−19.11	−47.09
Complex 4	−158.42	−196.80	325.95	−16.32	−45.59

## Data Availability

Not applicable.

## References

[1] Lavanchy D. (2005). Worldwide epidemiology of hbv infection, disease burden, and vaccine prevention. J. Clin. Virol..

[2] Henkler F., Hoare J., Waseem N., Goldin R.D., McGarvey M.J., Koshy R., King I.A. (2001). Intracellular localization of the hepatitis b virus hbx protein. J. Gen. Virol..

[3] Benn J., Schneider R.J. (1995). Hepatitis b virus hbx protein deregulates cell cycle checkpoint controls. Proc. Natl. Acad. Sci. USA.

[4] Hayes C.N., Akamatsu S., Tsuge M., Miki D., Akiyama R., Abe H., Ochi H., Hiraga N., Imamura M., Takahashi S. (2012). Hepatitis b virus-specific mirnas and argonaute2 play a role in the viral life cycle. PLoS ONE.

[5] Sekiba K., Otsuka M., Ohno M., Yamagami M., Kishikawa T., Suzuki T., Ishibashi R., Seimiya T., Tanaka E., Koike K. (2019). Inhibition of hbv transcription from cccdna with nitazoxanide by targeting the hbx–ddb1 interaction. Cell. Mol. Gastroenterol. Hepatol..

[6] Livingston C.M., Ramakrishnan D., Strubin M., Fletcher S.P., Beran R.K. (2017). Identifying and characterizing interplay between hepatitis b virus x protein and smc5/6. Viruses.

[7] Morikawa K., Suda G., Sakamoto N. (2016). Viral life cycle of hepatitis b virus: Host factors and druggable targets. Hepatol. Res..

[8] Jiang T., Liu M., Wu J., Shi Y. (2016). Structural and biochemical analysis of bcl-2 interaction with the hepatitis b virus protein hbx. Proc. Natl. Acad. Sci. USA.

[9] Miao J., Chen G.G., Chun S.-Y., Lai P.P. (2006). Hepatitis b virus x protein induces apoptosis in hepatoma cells through inhibiting bcl-xl expression. Cancer Lett..

[10] Kusunoki H., Tanaka T., Kohno T., Wakamatsu K., Hamaguchi I. (2014). Structural characterization of the bh3-like motif of hepatitis b virus x protein. Biochem. Biophys. Res. Commun..

[11] Kusunoki H., Tanaka T., Kohno T., Kimura H., Hosoda K., Wakamatsu K., Hamaguchi I. (2017). Expression, purification and characterization of hepatitis b virus x protein bh3-like motif-linker-bcl-xl fusion protein for structural studies. Biochem. Biophys. Rep..

[12] Zhang T.-Y., Chen H.-Y., Cao J.-L., Xiong H.-L., Mo X.-B., Li T.-L., Kang X.-Z., Zhao J.-H., Yin B., Zhao X. (2019). Structural and functional analyses of hepatitis b virus x protein bh3-like domain and bcl-xl interaction. Nat. Commun..

[13] Kim D.E., Chivian D., Baker D. (2004). Protein structure prediction and analysis using the robetta server. Nucleic Acids Res..

[14] Heo L., Park H., Seok C. (2013). Galaxyrefine: Protein structure refinement driven by side-chain repacking. Nucleic Acids Res..

[15] Wiederstein M., Sippl M.J. (2007). Prosa-web: Interactive web service for the recognition of errors in three-dimensional structures of proteins. Nucleic Acids Res..

[16] Junaid M., Almuqri E.A., Liu J., Zhang H. (2016). Analyses of the binding between water soluble c60 derivatives and potential drug targets through a molecular docking approach. PLoS ONE.

[17] Dallakyan S., Olson A.J. (2015). Small-molecule library screening by docking with pyrx. Chemical Biology.

[18] Ravindranath P.A., Forli S., Goodsell D.S., Olson A.J., Sanner M.F. (2015). Autodockfr: Advances in protein-ligand docking with explicitly specified binding site flexibility. PLoS Comput. Biol..

[19] Case D.A., Cheatham T.E., Darden T., Gohlke H., Luo R., Merz K.M., Onufriev A., Simmerling C., Wang B., Woods R.J. (2005). The amber biomolecular simulation programs. J. Comput. Chem..

[20] Price D.J., Brooks C.L. (2004). A modified tip3p water potential for simulation with ewald summation. J. Chem. Phys..

[21] Roe D.R., Cheatham T.E. (2013). Ptraj and cpptraj: Software for processing and analysis of molecular dynamics trajectory data. J. Chem. Theory Comput..

[22] Sun H., Li Y., Tian S., Xu L., Hou T. (2014). Assessing the performance of mm/pbsa and mm/gbsa methods. 4. Accuracies of mm/pbsa and mm/gbsa methodologies evaluated by various simulation protocols using pdbbind data set. Phys. Chem. Chem. Phys..

[23] Hou T., Li N., Li Y., Wang W. (2012). Characterization of domain–peptide interaction interface: Prediction of sh3 domain-mediated protein–protein interaction network in yeast by generic structure-based models. J. Proteome Res..

[24] Chen F., Liu H., Sun H., Pan P., Li Y., Li D., Hou T. (2016). Assessing the performance of the mm/pbsa and mm/gbsa methods. 6. Capability to predict protein–protein binding free energies and re-rank binding poses generated by protein–protein docking. Phys. Chem. Chem. Phys..

[25] Miller B.R., McGee T.D., Swails J.M., Homeyer N., Gohlke H., Roitberg A.E. (2012). Mmpbsa. Py: An efficient program for end-state free energy calculations. J. Chem. Theory Comput..

[26] Wang Y., Khan A., Chandra Kaushik A., Junaid M., Zhang X., Wei D.-Q. (2019). The systematic modeling studies and free energy calculations of the phenazine compounds as anti-tuberculosis agents. J. Biomol. Struct. Dyn..

[27] Khan A., Kaushik A.C., Ali S.S., Ahmad N., Wei D.-Q. (2019). Deep-learning-based target screening and similarity search for the predicted inhibitors of the pathways in parkinson’s disease. RSC Adv..

[28] Humayun F., Khan A., Ahmad S., Yuchen W., Wei G., Nizam-Uddin N., Hussain Z., Khan W., Zaman N., Rizwan M. (2021). Abrogation of SARS-CoV-2 interaction with host (nrp1) neuropilin-1 receptor through high-affinity marine natural compounds to curtail the infectivity: A structural-dynamics data. Comput. Biol. Med..

[29] Khan A., Ali S.S., Khan M.T., Saleem S., Ali A., Suleman M., Babar Z., Shafiq A., Khan M., Wei D.-Q. (2021). Combined drug repurposing and virtual screening strategies with molecular dynamics simulation identified potent inhibitors for SARS-CoV-2 main protease (3clpro). J. Biomol. Struct. Dyn..

[30] Khan A., Khan M., Saleem S., Babar Z., Ali A., Khan A.A., Sardar Z., Hamayun F., Ali S.S., Wei D.-Q. (2020). Phylogenetic analysis and structural perspectives of rna-dependent rna-polymerase inhibition from SARS-CoV-2 with natural products. Interdiscip. Sci. Comput. Life Sci..

[31] Xue L.C., Rodrigues J.P., Kastritis P.L., Bonvin A.M., Vangone A. (2016). Prodigy: A web server for predicting the binding affinity of protein–protein complexes. Bioinformatics.

